# Prostate-specific membrane antigen expression predicts recurrence of papillary thyroid carcinoma after total thyroidectomy

**DOI:** 10.1186/s12885-022-10375-z

**Published:** 2022-12-07

**Authors:** Young Jae Ryu, Soo Young Lim, Yong Min Na, Min Ho Park, Seong Young Kwon, Ji Shin Lee

**Affiliations:** 1 Department of Surgery, Chonnam National University Medical School and Hwasun Hospital, Hwasun-gun, Jeonnam Republic of Korea; 2 Department of Nuclear Medicine, Chonnam National University Medical School and Hwasun Hospital, Hwasun-gun, Jeonnam Republic of Korea; 3 Department of Nuclear Medicine, Chonnam National University Medical School and Hwasun Hospital, 322 Seoyang-ro, Hwasun-gun, Jeonnam 58128 Republic of Korea; 4 Department of Pathology, Chonnam National University Medical School and Hwasun Hospital, Hwasun-gun, Jeonnam Republic of Korea; 5 Department of Pathology, Chonnam National University Medical School and Hwasun Hospital, 322 Seoyang-ro, Hwasun-gun, Jeonnam 58128 Republic of Korea

**Keywords:** Glutamate carboxypeptidase II, Thyroid neoplasm, Immunohistochemistry, Endothelial neovasculature

## Abstract

**Background:**

Prostate-specific membrane antigen (PSMA) overexpression has been observed in the endothelial neovasculature of several solid malignancies. This study aimed to identify PSMA expression in the primary tumor of classical papillary thyroid carcinoma (PTC) and assess the correlation between the degree of PSMA expression and recurrence.

**Methods:**

We reviewed the electronic medical records of patients who underwent total thyroidectomy and central neck dissection, with or without lateral neck dissection, for classical PTC between 2009 and 2014 at our institution. Recurrence was defined as a structural disease based on histological confirmation on follow-up. Fifty-one patients with the recurrent structural disease were matched, using a propensity score matching method, to patients with no disease evidence during follow-up. Clinicopathological and follow-up data were collected for 102 patients. The monoclonal mouse anti-human PSMA/FOLH1/NAALADase I antibody was used for staining the primary tumor. The score of PSMA expression was classified as negative (< 5% positivity), weak (5–10 % positivity), moderate (11–49% positivity), and strong (more than 50% positivity). Clinicopathological factors were compared between patients with low and high PSMA expression. Moreover, whether the degree of PSMA expression and clinicopathological factors could predict recurrence was investigated. Cox proportional hazard regression models were used to evaluate the risk of recurrence.

**Results:**

There was no significant difference in clinicopathological factors between low (negative or weak) and high (moderate or strong) PSMA expression. Gross extrathyroidal extension (ETE), absence of chronic lymphocytic thyroiditis, and high PSMA expression were all associated with lower recurrence-free survival (RFS) rate in a univariate analysis. In multivariate analysis, gross ETE (hazard ratio [HR], 2.279; 95% confidence interval [CI], 1.257−4.132; *p* = 0.007) and high PSMA expression (HR, 1.895; 95% CI, 1.073−3.348; *p* = 0.028) were associated with poor RFS.

**Conclusions:**

High PSMA expression in the primary tumor was a significant factor in predicting recurrence in classic PTC. PSMA could be a potential biomarker for personalized management for PTC.

## Background

Papillary thyroid carcinoma (PTC) accounts for up to 85% of thyroid malignancies, and its incidence has been increasing for decades [1, 2]. PTC has an indolent course and better survival with main treatment strategies including thyroidectomy, radioactive iodine (RAI) therapy, and thyroid stimulating hormone (TSH) suppression. Nevertheless, up to 15% of differentiated thyroid carcinoma (DTC) become RAI-refractory thyroid cancer, and 10% develop distant organic metastases [3]. RAI-refractory thyroid cancer can be assessed using ^18^F-fluorodeoxyglucose (FDG) positron emission tomography with computed tomography (PET-CT). However, the intensity of ^18^F-FDG uptake reportedly does not predict the progression of differentiated metastatic thyroid tumors [4]. Moreover, the management of RAI-refractory thyroid cancer remains challenging because of the severe adverse effects of tyrosine kinase inhibitors.

Pathologic neovascular formation, which is correlated with aggressive tumor growth and distant metastasis, could be a significant target for imaging studies and cancer management. Prostate-specific membrane antigen (PSMA), also known as glutamate carboxypeptidase II or folate hydrolase 1, is found to be overexpressed in prostate carcinoma epithelium. Beyond prostatic carcinoma, several studies have revealed the overexpression of PSMA in the endothelial neovasculature of various solid malignancies [5–8]. Endothelial cells play an important role in tumor cell intravasation and extravasation. Therefore, identifying PSMA expression in tumor cell endothelium during the preoperative evaluation will be helpful in verifying the extent of the primary tumor and the presence of distant metastases, including skip lesions. After the first report by Verburg et al. regarding PSMA expression in DTC patients with negative RAI scan [9], several studies have demonstrated that ^68^Ga-PSMA PET-CT might be superior to ^18^F-FDG PET-CT for structural disease visualization [10, 11]. High PSMA expression in DTC can predict tumor aggressiveness and RAI-refractoriness [12].

Excluding advanced DTC, information on PSMA expression in PTC may be helpful in decision-making regarding postoperative management. Moreover, additional imaging modalities using PSMA-based techniques may expand the treatment options and extent of surgical operations for patients with recurrent malignancies. However, research on the relationship between PSMA expression in primary tumor neovasculature and prognosis in classical PTC is insufficient. Therefore, this study aimed to identify PSMA expression in the primary tumor of classical PTC and investigate the impact of PSMA expression on recurrence.

## Methods

### Study population

We reviewed the electronic medical records of patients who underwent central neck dissection with or without lateral neck dissection, including total thyroidectomy for PTC between 2009 and 2014 at Chonnam National University Hwasun Hospital. Patients who had a suspicious residual or persistent disease, accompanying distant metastases at initial diagnosis, and secondary primary cancer throughout the follow-up period were excluded. The institutional review board of Chonnam National University Hwasun Hospital approved this study (approval no. CNUHH-2019-237) and waived the requirement of obtaining informed consent due to the retrospective study design.

### Initial therapy

Thyroid nodules of the enrolled patients were evaluated by fine-needle aspiration cytology (FNAC) and classified as category V or VI using the Bethesda system [13]. Cervical lymph nodes (LN) were preoperatively assessed using neck ultrasonography (US) and enhanced CT. FNAC was conducted for lateral cervical LN with suspicious characteristics, such as the absence of an echogenic hilum, irregular margin, round shape, cystic aspect, peripheral vascularity, and hyperechogenicity. The enrolled patients underwent total thyroidectomy. Patients who tested clinically negative for LN underwent prophylactic central neck dissection. Compartment-oriented lateral neck dissection (from II to V) was performed in patients with metastatic lateral neck LN. RAI therapy was performed 2−3 months after surgery for any patient with gross extrathyroidal extension (ETE) or a large LN involvement burden.

### Follow-up

All the patients underwent routine physical examination, including assessment of triiodothyronine, free thyroxine, TSH, thyroglobulin (Tg), anti-Tg antibody, and neck US every 3−6 months after the initial treatment. A post-therapeutic whole-body scan was obtained one or two years after surgery. PET-CT scans were performed for patients who had increased Tg levels with negative US and RAI scans. The interval between regular follow-up examination was extended for patients who showed an excellent response. Recurrence was defined as a structural disease based on histological confirmation. However, patients with a suspicious structural disease in an inaccessible distant organ were confirmed to have recurrence based on imaging studies.

### Immunohistochemical staining

Monoclonal mouse anti-human PSMA/FOLH1/NAALADase I antibody (clone 3E6, Novus Biologicals USA) was used to stain the classical PTC primary tumor [14]. The most representative and well-preserved tissue from an individual tumor was selected for immunohistochemical staining of the formalin-fixed, paraffin-embedded whole tissue. PSMA expression in the specimen was evaluated more than twice by experienced pathologists. The score of PSMA expression was classified as negative (< 5% positivity), weak (5–10 % positivity), moderate (11–49% positivity), and strong (≥ 50% of positivity). Finally, low PSMA expression was defined as a score including negative or weak and high PSMA expression was defined as a score including moderate or strong.

### Data analysis

A total of 51 patients with the recurrent structural disease were matched according to age and sex, using a propensity score matching method, to patients with no disease evidence during follow-up. In addition, clinicopathological and follow-up data were collected for 102 patients. We compared clinicopathological factors between low and high PSMA expression and evaluated whether the degree of PSMA expression as well as clinicopathological factors could predict recurrence. T-stage, N-stage, and gross ETE were based on the American Joint Committee on Cancer (AJCC) 8^th^ edition [15].

### Statistical analysis

The mean (± standard deviation, SD) and independent sample t-test were used for normally distributed continuous variables. In contrast, the median (interquartile range) and Mann-Whitney U test were used for non-normally distributed continuous variables. Categorical variables are presented as a value (percent) and compared using chi-square or Fisher’s exact tests. Recurrence-free survival (RFS) was defined as the interval between the initial surgery and the confirmation of structural recurrence. Univariate and multivariate Cox proportional hazard regression models were used to evaluate the risk of recurrence. The SPSS 26.0 software was used for all statistical analyses. A *p-*value of <0.05 was considered statistically significant.

## Results

### Patients’ demographics

Of the 102 patients in this study, 19 (18.6%) were older than 55 years, and 34 (33.3%) were men. The median tumor size was 1.2 cm, with 38 patients (37.3%) having microcarcinoma as the primary tumor of ≤1 cm. Tumor multiplicity and bilaterality were noted in 28 (27.5%) and 21 (20.6%) patients, respectively. Overall, 22 patients had no LN involvement; in contrast, N1a status was noted in 41 (40.2%) patients and N1b in 39 (38.2%) patients. A total of 23 (22.5%) patients had gross ETE, with the most common stage being stage I (83.3%). The median follow-up was 55.5 months (range, 13–122 months) (Table 1).

**Table 1:** Patients' demographics (*n* = 102)

Variables	Value (% or range)
Age (mean, standard deviation)	43.7 years, 12.4
<55 years/ ≥55 years	83 (81.4)/ 19 (18.6)
Female/ Male	68 (66.7)/ 34 (33.3)
Tumor size (median, interquartile range)	1.2 cm, 0.9
≤1cm/ >1cm	38 (37.3)/ 64 (62.7)
T stage	
T1a/ T1b / T2	38 (37.3)/ 31 (30.4)/ 10 (9.8)
T3a/ T3b/ T4a	0/ 9 (8.8)/ 14 (13.7)
Multiplicity	28 (27.5)
Bilaterality	21 (20.6)
N stage	
N0/ N1a/ N1b	22 (21.6)/ 41 (40.2)/ 39 (38.2)
Chronic lymphocytic thyroiditis	32 (31.4)
Gross extrathyroidal extension	23 (22.5)
Stage	
I/ II/ III	85 (83.3)/ 11 (10.8)/ 6 (5.9)
PSMA expression	
Negative/ Weak	31 (30.4)/ 20 (19.6)
Moderate/ Strong	20 (19.6)/ 31 (30.4)
Radioactive iodine therapy	85 (83.3)
Recurrence	51 (50.0))
Central neck	11 (10.8)
Central neck plus lateral neck	8 (7.8)
Lateral neck	29 (28.4)
Lung	2 (2.0)
Lung plus central and lateral neck	1 (1.0)
Median follow up period	55.5 months (range, 13–122 months)

*PSMA* prostate-specific membrane antigen

### Comparison between low and high PSMA expression

**Fig. 1:**
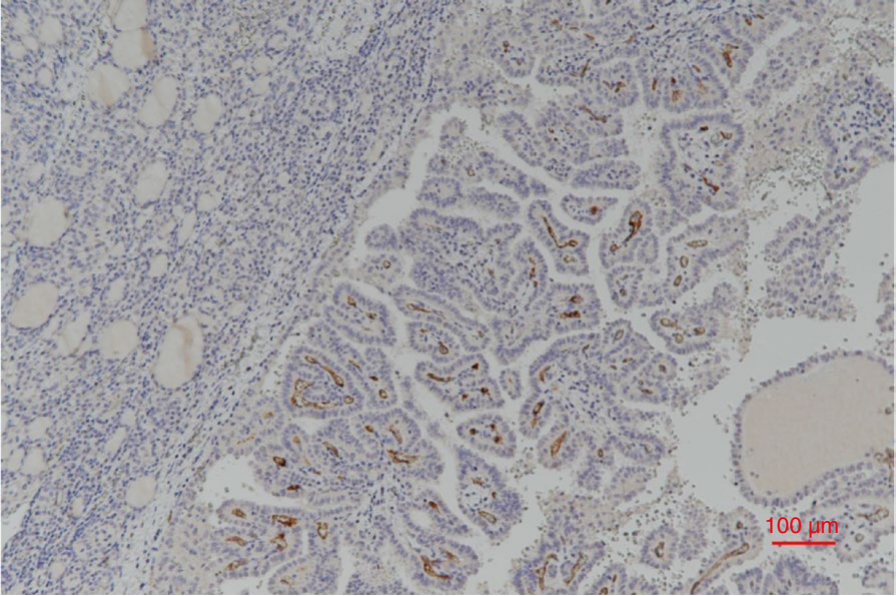
Prostate-specific membrane antigen expression in normal thyroid (left) and papillary thyroid carcinoma (right) (x 100)

**Fig. 2:**
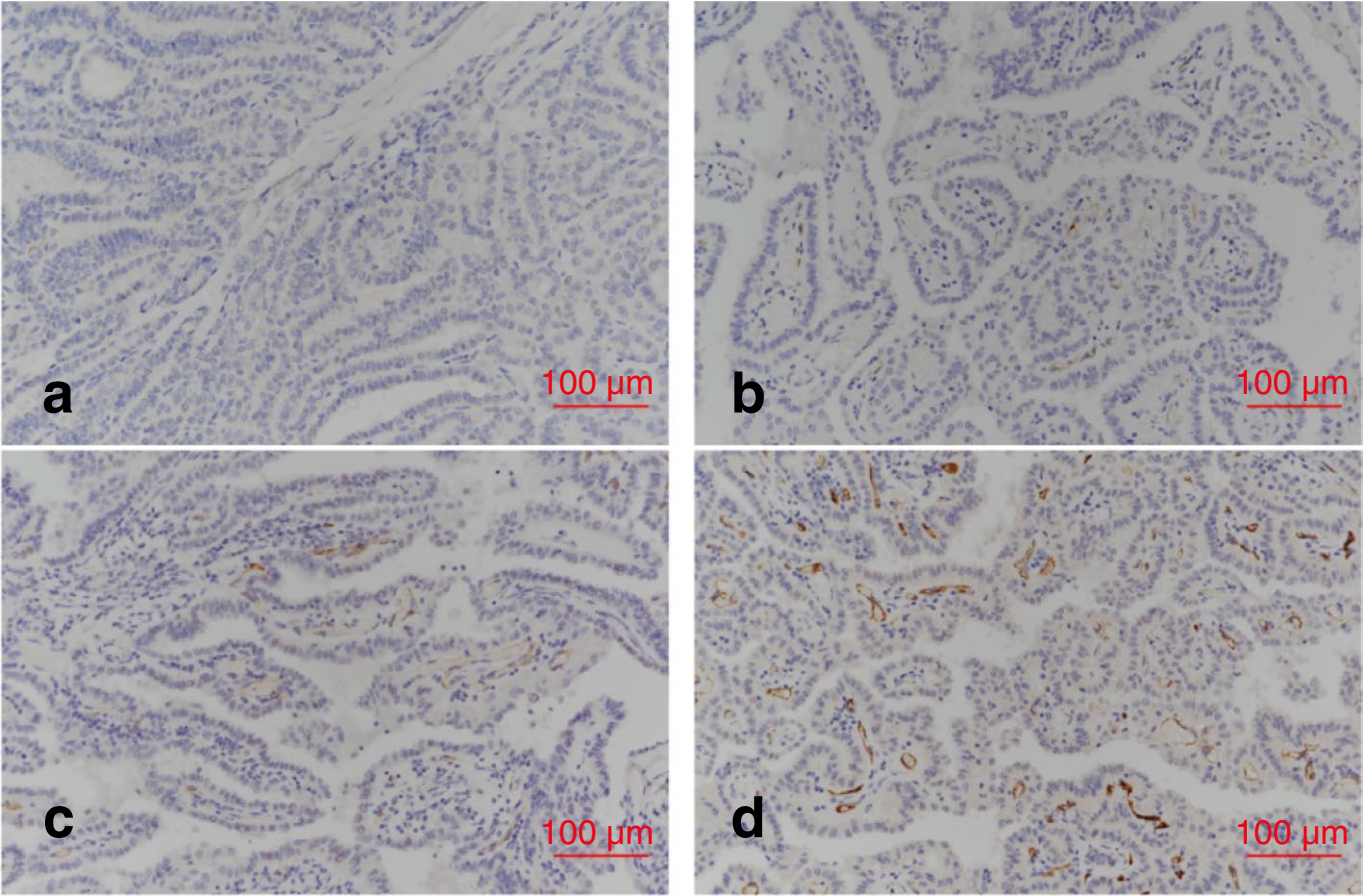
Prostate-specific membrane antigen expression, negative (**a**), weak (**b**), moderate (**c**), strong (**d**) (x 200)

Figure 1 shows the difference in PSMA expression between normal tissue and tumor lesions. The different PSMA expression patterns are shown in Fig. 2. Clinicopathologic factors were compared between low (negative, weak) and high (moderate, strong) PSMA expression. There were no significant differences in sex, age, tumor size, multiplicity, bilaterality, chronic lymphocytic thyroiditis (CLT), gross ETE, or LN involvement between low and high PSMA expression (Table 2).

**Table 2:** Comparison according to PSMA expression

	Low PSMA	High PSMA	*p* value
Sex			0.529
Female	32 (62.7)	36 (70.6)	
Male	19 (37.3)	15 (29.4)	
Age			0.612
<55 years	40 (78.4)	43 (84.3)	
≥55 years	11 (21.6)	8 (15.7)	
Tumor size			0.151
≤1 cm	23 (45.1)	15 (29.4)	
>1 cm	28 (54.9)	36 (70.6)	
Multiplicity			0.825
Absence	36 (70.6)	38 (74.5)	
Presence	15 (29.4)	13 (25.5)	
Bilaterality			0.625
Absence	39 (76.5)	42 (82.4)	
Presence	12 (23.5)	9 (17.6)	
Chronic lymphocytic thyroiditis			0.831
Absence	34 (66.7)	36 (70.6)	
Presence	17 (33.3)	15 (29.4)	
Gross extrathyroidal extension			0.636
Absence	41 (80.4)	38 (74.5)	
Presence	10 (19.6)	13 (25.5)	
Lymph node involvement			0.228
Absence	14 (27.5)	8 (15.7)	
Presence	37 (72.5)	43 (84.3)	
N stage			0.321
N0	14 (27.5)	8 (15.7)	
N1a	18 (35.3)	23 (45.1)	
N1b	19 (37.3)	20 (39.2)	
Radioactive iodine therapy	41 (80.4)	44 (86.3)	0.596

*PSMA* prostate-specific membrane antigen

### Univariate and multivariate analysis of recurrence

In univariate recurrence analysis, gross ETE, CLT absence, and high PSMA expression were associated with worse RFS. However, there were no significant differences in age, sex, tumor size, tumor multiplicity and bilaterality, LN involvement, or stage (Table 3). In multivariate analysis, gross ETE (HR, 2.279; 95% CI, 1.257−4.132; *p* = 0.007) and high PSMA (HR, 1.895; 95% CI, 1.073−3.348; *p* = 0.028) expression were associated with poor RFS (Table 4).

**Table 3:** Univariate Cox regression of recurrence

Variables	Reference	HR (95% CI)	*p* value
Age (≥55 years )	<55 years	1.640 (0.858−3.135)	0.134
Sex (Male)	Female	0.890 (0.487−1.626)	0.704
Tumor size (>1cm)	≤1cm	1.451 (0.794−2.652)	0.227
Multiplicity (Presence)	Absence	1.484 (0.829−2.658)	0.184
Bilaterality (Presence)	Absence	0.934 (0.468−1.864)	0.846
CLT (Presence)	Absence	0.507 (0.260−0.989)	0.046
Gross ETE (Presence)	Absence	2.267 (1.251−4.107)	0.007
LN involvement (Presence)	Absence	2.236 (0.953−5.246)	0.064
Stage (II/III)	I	1.352 (0.677−2.700)	0.392
PSMA (High)	Low	1.887 (1.069−3.331)	0.029

*CLT* chronic lymphocytic thyroiditis, *ETE* extrathyroidal extension, *LN* lymph node, *PSMA* prostate-specific membrane antigen

**Table 4:** Multivariate Cox regression of recurrence

Variables	Reference	HR (95% CI)	*p* value
Age (≥55 years)	<55 years	1.314 (0.662−2.608)	0.435
CLT (Presence)	Absence	0.581 (0.295−1.145)	0.117
Gross ETE (Presence)	Absence	2.279 (1.257−4.132)	0.007
LN involvement (Presence)	Absence	1.638 (0.671−4.000)	0.279
PSMA (High)	Low	1.895 (1.073−3.348)	0.028

*CLT* chronic lymphocytic thyroiditis, *ETE* extrathyroidal extension, *LN* lymph node, *PSMA* prostate-specific membrane antigen

## Discussion

This study identified PSMA expression in classical PTC and examined the correlation between PSMA expression and recurrence. We found that the degree of PSMA expression in PTC endothelial neovasculature is inhomogeneous, like other solid malignancies. Although there was no definitive link between the PSMA expression levels and clinicopathological variables in PTC, PTC patients with high PSMA expression had a higher likelihood of recurrence than those with low PSMA expression.

Since the introduction of PSMA cloning as a technique for identifying a prostate cancer biomarker by Israeli et al. in 1993 [16], increasing research on the role of PSMA in various malignancies have been conducted. PSMA, also known as glutamate carboxypeptidase II, N-acetyl-L-aspartyl-L-glutamate peptidase I (NAALADase I) or folate hydrolase 1, is a type II integral membrane protein [17]. It has a large extracellular region that induces the internalization sequence and peptidase activities involved in tumor cell pro-drug targeting [18]. As the name implies, PSMA is expressed in the prostate gland epithelium; when overexpressed, as it is in malignancies, its levels of expression are used in disease staging and management. Several recent studies have found that PSMA is overexpressed in the vascular endothelium of various solid malignancies, such as lung, breast, renal, and urothelial tumors [5–8]. Based on physiological conditions, new vessel formation is regulated by cellular (co-option, intussusception, angiogenesis, and vasculogenesis) and molecular mechanisms [19, 20]. As the formation of neovasculature is an essential factor for tumor growth and invasiveness, PSMA expression in neovasculature is an emerging marker used in diagnosing and managemet of cancer.

Although no consensus was reached for a standardized interpretation of PSMA immunohistochemical staining results, several findings, such as the intensity of staining, percentage of positive cells, and the absolute number of PSMA-positive microvessels, were used instead [21, 22]. PSMA expression varies widely among different subtypes of thyroid carcinoma. PSMA expression in poorly differentiated thyroid carcinoma or undifferentiated thyroid carcinoma is higher than that in normal thyroid tissue or benign thyroid tumors, such as follicular adenoma [23]. One study demonstrated that all tissues, such as those in classical PTC, follicular thyroid carcinoma, and distant metastatic lesions showed PSMA expression despite differences in the degree of expression [22]. Furthermore, DTC patients with persistent or recurrent disease in the thyroidectomy bed or LN showed PSMA expression [24, 25]. Bychkov et al. found that a larger tumor size in DTC is significantly associated with a higher positivity of PSMA expression and that PSMA expression level in lymphoid follicle dendritic cells accompanying Hashimoto’s thyroiditis is similar to that in metastatic cervical LN [26]. The present study assessed PSMA expression using a simple method in primary tumors, but not in LNs or distant metastases. PSMA expression was present in 72.5% and 66.7% of patients with classic PTC, according to the presence and absence of recurrence. There were no significant differences in the degree of PSMA expression and clinicopathological factors. However, high PSMA expression was associated with unfavorable RFS. Therefore, high PSMA expression may be an indicator for predicting recurrence.

Several studies revealed the relationship between the degree of PSMA expression and the uptake of PSMA PET-CT in solid malignancies [27–29]. PSMA uptake is not influenced by sodium iodide symporter. Therefore, PSMA-based imaging is free from the TSH stimulation method for PTC evaluation. Evaluation of PSMA expression in the initial surgical DTC specimen could be helpful for RAI-refractory or de-differentiation prediction [30]. One study has suggested that ^68^Ga-PSMA PET-CT could be better than ^18^F-FDG PET-CT in identifying structural diseases and enabling PSMA-targeted radionuclide therapy in patients who had metastasized and RAI-negative DTC [31]. Although the amount of vascularization might be affected by PSMA expression heterogeneity, ^177^Lu-PSMA-617 therapy based on the results of ^68^Ga-PSMA PET-CT reportedly presented a mild temporary response in patients with RAI-refractory DTC [32]. The present study showed that high PSMA expression in the primary tumor in PTC was associated with a short RFS. Therefore, PSMA PET-CT could apply to the detection of recurrence or distant metastases after initial surgery if the primary tumor of PTC has high PSMA expression. However, further studies are needed for precise management according to the degree of PSMA expression.

PSMA expression has been shown to positively impact predicting survival outcomes for prostatic carcinoma [33, 34]. In colorectal carcinoma, the amount of PSMA expression is positively correlated with histologic grade; however, no significant relationship has been reported between PSMA expression and survival outcomes [5]. In contrast, as reported previously, patients with high PSMA expression had worse survival rates than those with low PSMA expression in oral squamous cell carcinoma [6]. The patients with medullary thyroid carcinoma have different tumor progression according to the PSMA value [35]. Surgery, RAI therapy, or TSH suppression may help most patients with PTC attain a good prognosis. However, some patients exhibited incomplete biochemical responses during the follow-up period. PSMA can be a promising biomarker to supplement the BRAF^v600E^ mutation or telomerase reverse transcriptase (TERT) promoter mutations to predict prognosis. Moreover, PSMA-based imaging modalities may provide the opportunity to acquire more information for patients planning active surveillance because preoperative PSMA-based imaging findings are expected to predict the recurrence after initial management. Although PSMA expression was easily acquired using immunohistochemical staining, it was not specific to thyroid carcinoma [36]. Careful selection for PSMA-based imaging is required during the initial phase of PTC. Therefore, investigating PSMA expression in sample tissues from diagnosis would help in designing personalized treatment strategies and predicting recurrence.

This study has several limitations. First, the study analysis was retrospective and included only a small number of patients. Second, only structural diseases were labeled as true recurrence; therefore, incomplete biochemical and indeterminate responses were not accounted for. Third, oncogenic mutations, such as BRAF^v600E^ and the TERT promoter, were not included in the present study. Fourth, PSMA expression was only evaluated in the primary tumor of the thyroid gland. Further studies using satisfactory data are required to overcome these limitations.

## Conclusions

Various degrees of PSMA expressions were observed in the primary tumor of PTC. Although there was no significant difference in clinicopathological factors according to low (negative and weak) and high (moderate and strong) PSMA expression, high PSMA expression in the primary tumor was a significant factor in predicting recurrence in classical PTC. PSMA could be a potential biomarker for predicting disease progression and designing personalized management for PTC.

## Data Availability

The datasets used and/or analysed during the current study are available from the corresponding author on reasonable request.

## Abbreviations

DTC: Differentiated thyroid carcinoma
FDG: Fluorodeoxyglucose
ETE: Extrathyroidal extension
FNAC: Fine-needle aspiration cytology
LN: Lymph nodes
PTC: Papillary thyroid carcinoma
PET-CT: Positron emission tomography with computed tomography
PSMA: Prostate-specific membrane antigen
RAI: Radioactive iodine
RFS: Recurrence-free survival
TERT: Telomerase reverse transcriptase
TSH: Thyroid stimulating hormone
US: Ultrasonography
